# Antisocial Behavior in Cooperative Bacteria (or, Why Can't Bacteria Just Get Along?)

**DOI:** 10.1371/journal.pbio.0030398

**Published:** 2005-11-01

Bacteria are defined as unicellular organisms, but they don't typically function as single cells in nature. Social behavior among bacteria is well established, and makes a lot of sense when you consider that billions of bacteria—representing as many as 1,000,000 species—can be found in just one gram of fertile soil. Cooperative bacteria coordinate a range of complex behaviors through a density-dependent mechanism called quorum sensing: when bacterial numbers reach a critical mass, individual cells secrete signaling molecules that control the behavior of the colony. Through quorum sensing, individual cells amass into biofilms (bacterial colonies that exude slime and other molecules that help them stick to everything from ship hulls to teeth), and some species are able to form structures called fruiting bodies to weather nutrient-poor conditions.

One group of bacterial species, known as the myxobacteria, exhibit several sophisticated social behaviors. They socially swarm and hunt other microbes in a manner analogous to wolf pack hunting. Even more dramatically, when cells of the species Myxococcus xanthus fall upon hard times due to lack of food, some 100,000 individuals band together and form fruiting structures. This process is marked by distinct gene expression programs, differentiation, and morphological changes. Inside the fruiting body, rod-shaped cells differentiate into spherical, stress-resistant spores designed to wait out a famine. But only a portion of the population turns into spores; the vast majority either commit cell suicide, making the ultimate sacrifice, or remain undifferentiated.

But how far does this cooperative behavior go? One species of bacteria can comprise many divergent strains, with different genotypes. It's been shown that when two distinct Myxococcus species are mixed together, the species segregate and form separate fruiting bodies, with one species dominating the other in spore production. Would mixing divergent strains of the same species produce similar results? In a new study, Francesca Fiegna and Gregory Velicer investigated this question using nine strains of the “highly social” ubiquitous soil bacterium M. xanthus isolated from different regions of the world.

To see how divergent strains behave in mixed company, Fiegna and Velicer placed the divergent strains in nutrient-poor cultures, pitting every possible combination of one strain against another. After starving the mixed cultures for five days, the authors observed each pair's fruiting body formation, as well as the spore production of each strain in the mixtures and in isolation. [Fig pbio-0030398-g001]

**Figure pbio-0030398-g001:**
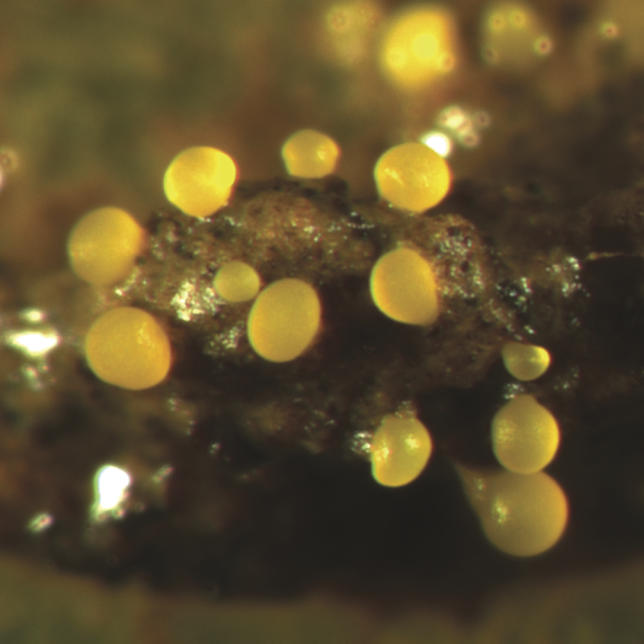
Some 100,000 Myxococcus xanthus cells amassed into a fruiting body with spores, above. Experimental competitions showed that some strains of this social bacterium exploited others. (Image: Michiel Vos)

The shape, size, and distribution of fruiting bodies were different for nearly every mixed pair relative to their clonal cultures, with most pairs producing fewer fruiting bodies than each strain in isolation. Mixing also decreased the overall social productivity (indicated by total spore production) of the pairs, with some antagonistic pairs reducing total spore production as much as 90%. Even though most strains responded poorly to mixing, some performed better in competition than in isolation—revealing that naturally occurring social bacteria are capable of exploiting their neighbors.

Fiegna and Velicer went on to rank the dominant strains (that is, determined which strain produced the most spores), based on the possible pairing interactions, and showed that their fitness ratings were largely hierarchical, with only one case of a rock-paper-scissors (circular) fitness relationship among any three strains out of 82 such comparisons. This hierarchy suggests that diversity would be quickly lost if all nine strains resided together in one mixed population, with only one strain (or a small number of strains) dominating and eliminating the others over time. Thus, these strains do not tend to act as cooperative subunits when mixed, and M. xanthus as a species has diverged into multiple, distinct social types that cooperate with clone-mates (and perhaps close relatives) but have no qualms about exploiting distant relatives of the same species.

Since M. xanthus can travel great distances carried by water, wind, and an array of animals and insects, the authors conclude, it's possible that resulting antagonisms between introduced foreign strains and resident bacterial populations might decimate some native populations. The degree to which this type of mixing occurs in nature is an active area of research. With the help of whole-genome sequencing and molecular techniques, scientists can refine their traditional morphological classifications of this social soil bacterium to better understand its distribution and likely encounters in soil communities—whether the fitness hierarchies seen here are more typical of mixed distant rather than local strains, for example—and to begin unraveling the molecular agents of subjugation. —*Liza Gross*

